# Circular active noise barrier using theoretical control filter considering interaction between speaker and barrier

**DOI:** 10.1038/s41598-023-27756-4

**Published:** 2023-02-14

**Authors:** Sanghyeon Lee, Youngjin Park

**Affiliations:** Korea Advanced Institute of Science and Technology (KAIST), Mechanical Engineering, 291 Daehak-ro, Yuseong-gu, Daejeon, 34141 Republic of Korea

**Keywords:** Mechanical engineering, Acoustics

## Abstract

A circular active noise barrier using a theoretically calculated control filter without real-time adaptation was proposed for noise reduction in a specific outdoor space. A compact circular barrier is used for the movable system to cope with changes in the location of the workspace, and noise in a wide frequency band can be reduced by conducting active noise control through control speakers arranged around a barrier. However, there was a significant performance gap compared with the maximum performance achieved by using the experimental fixed filter due to an extremely simplified theoretical model which ignores the interaction between the control speakers and the barrier. Therefore, this study tried to minimize the performance degradation when applying the theoretically calculated control filter. Another theoretical model is introduced to improve the noise reduction performance by considering the interaction between the control speaker and the barrier. Through experiment, it is confirmed that noise reduction performance is improved by about 2.6 dB in the frequency of interest.

## Introduction

Noise problems in industrial settings are becoming more various and serious, and coupled with increasingly stricter noise regulations, noise reduction is becoming a more important issue. Noise in various spaces such as rest areas or workspaces can lead to stress, distraction, and hearing loss^1^ and many workers in industries are exposed to loud noise for long periods of time. A noise barrier can be used to reduce noise, but it is ineffective for noise reduction in a low-frequency band due to the diffracted noise. In order to reinforce noise reduction in a low-frequency band, the active noise barrier^2–6^ that applies active noise control (ANC)^7^ to a barrier was studied. By placing speakers and error microphones above the barrier, the diffracted noise in a low-frequency band is reduced through ANC. The arrangement of microphones and speakers^8,9^, the method of obtaining the control filter^10,11^, and using unidirectional control sources^12^ have been studied to improve the performance of the active noise barrier. However, the active noise barrier using semi-infinite barrier is expensive and requires a large place to set up. In addition, it is hard to move, so it is applicable only to a fixed environment.

In order to solve this problem, the circular active noise barrier using a theoretically calculated control filter^13–15^ was proposed to reduce noise in a specific outdoor space instead of global noise reduction. The circular active noise barrier consists of a compact circular barrier and control speakers placed around the barrier with the aim of reducing noise in an individual target space such as a workspace or rest space. It requires less cost and space compared with the semi-infinite barrier, and can be moved and reinstalled. A theoretically calculated control filter without arranging microphones over the target space is used because the arranged microphones to apply ANC obstructs the workers. The theoretically calculated control filter can be calibrated quickly, so it is easy to respond to changes in the position of the noise source or the target control space.

However, the performance gap between the theoretically obtainable performance and the experimental results is significant. In the previous study^13^, a control filter is calculated based on an extremely simplified theoretical model aimed at providing a simply applicable noise control method. The performance degradation occurs due to the difference between the simplified theoretical model and the experimental system. Therefore, this study tried to minimize the performance degradation that occurs when the theoretically calculated control filter is applied to the circular hybrid noise control system. The interaction between the control speaker and the barrier, which is one of the main causes cannot be considered by the previous simplified theoretical model, so another theoretical model is introduced.

## Methods

### The circular active noise barrier and the theoretical model

**Figure 1:**
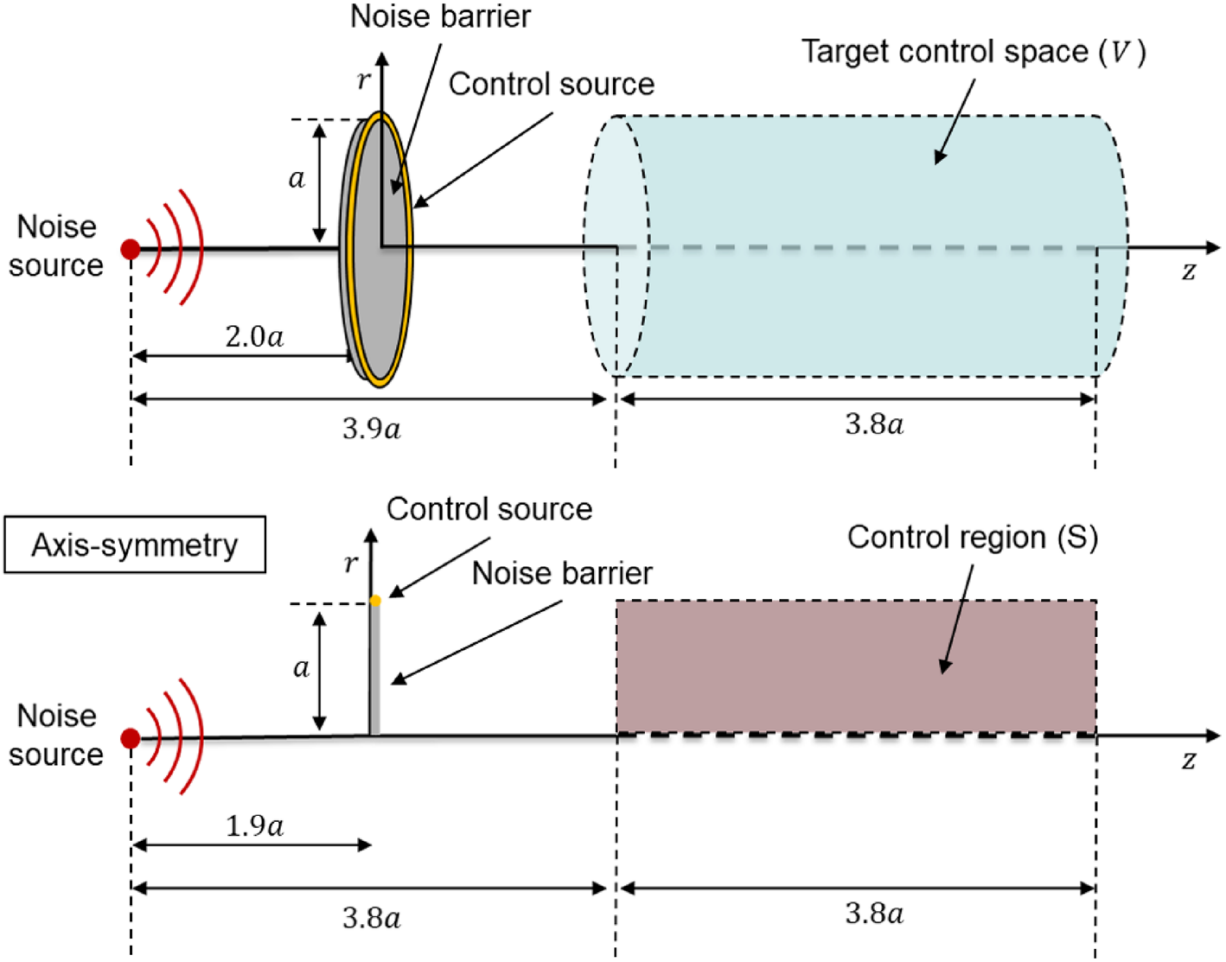
Configuration of the circular active noise barrier (top) and illustration of an axisymmetric structure in two-dimension (bottom). The control source is a circular line source in the prior study. *a* is the radius of the barrier. *V* is the target control space, and *S* is the cross-section of the axis-symmetric structure.

The circular active noise barrier is briefly described. The structure is shown in Fig. 1.A control filter for ANC is obtained to minimize the acoustic potential energy of the target control space as shown in Eq. (1).

1 $$\begin{aligned} \begin{aligned} C_w=\int _V \frac{|P_n-K_wP_c|^2}{2\rho c^2}dv \end{aligned} \end{aligned}$$

Here, $Pn \, [Pa]$ and $Pc \, [Pa]$ are respectively the pressure over the target control space by noise and the control source. *V* is the target control space, and *S* is the cross-section of the axis-symmetric structure. $\rho \, [kg/m^3]$ and $c \, [m/s]$ are the air density and speed of sound, respectively. The obtained control filter $K_w$ to minimize Eq. (1) is provided in Eq. (2).

2 $$\begin{aligned} \begin{aligned} K_{w,opt}=\Big {[}\int _VP_cP_ndv \Big {]} \Big {[}\int _V P_cP_cdv \Big {]}^{-1} \end{aligned} \end{aligned}$$

Because of the axisymmetric structure, the integral calculation domain for calculating the control filter can be changed from space to surface.

3 $$\begin{aligned} \begin{aligned} K_{w,opt}=\Big {[}\int _S P_cP_n rds \Big {]} \Big {[}\int _S P_cP_c rds \Big {]}^{-1} \end{aligned} \end{aligned}$$

The residual acoustic potential energy is given in Eq. (4).

4 $$\begin{aligned} \begin{aligned} C_{e}=\int _V \frac{|P_n-K_{w,opt}P_c|^2}{2\rho c^2}dv \end{aligned} \end{aligned}$$

Noise reduction is defined as the reduction of the acoustic potential energy in the target control space as shown in Eq. (5).

5 $$\begin{aligned} \begin{aligned} NR=10 \log _{10} \frac{C_{0}}{C_{e}} \end{aligned} \end{aligned}$$

$C_0$ is the acoustic potential energy in the target control space before reducing the noise $\big {(} C_{0}=\int _V \frac{|P_n|^2}{2\rho c^2}dv \big {)}$.

Noise reduction performance in the frequency band of interest is defined as delineated in Eq. (6).

6 $$\begin{aligned} \begin{aligned} NR_{performance}=10 \log _{10} \frac{C_{int,0}}{C_{int,e}} \end{aligned} \end{aligned}$$

$C_{int,0}$ is the summation of the $C_0$ in the frequency band of interest $\big {(}C_{int,0}=\int _F\ C_0 df \big {)}$. $C_{int,e}$ is the summation of the $C_e$ in the frequency band of interest $(C_{int,e}=\int _F C_e df )$. F is the frequency band of interest.

In order to obtain the control filter in Eq. (3), the theoretically calculated pressure is used. In the case of the noise, the equation established by Flammer^16^ is used by assuming the circular barrier as a very thin disk under an acoustically hard condition. The equation is written in the oblate spheroidal coordinate. The relations between the Cartesian coordinate (*x*, *y*, *z*) and the oblate spheroidal coordinate $(\xi ,\eta ,\phi )$ are given in Eq. (7).

7 $$\begin{aligned} \begin{aligned} x&=a\sqrt{(1-\eta ^2)(\xi ^2+1)}cos(\phi )\\ y&=a\sqrt{(1-\eta ^2)(\xi ^2+1)}sin(\phi )\\ z&=a\eta \xi \end{aligned} \end{aligned}$$

Here, *a* is the radius of a circular barrier. The pressure of noise located at $(\eta _0,\xi _0,\phi _0)$ with an acoustically hard circular barrier at the origin is shown in Eq. (8). The harmonic term $(e^{i \omega t})$ is omitted.

8 $$\begin{aligned} \begin{aligned} p_{noise}(\eta ,\xi ,\phi )&=\frac{ik}{2\pi } \sum ^{\infty }_{m=0}\sum ^{\infty }_{n=m} \frac{\varepsilon _m}{N_{mn}(-ika)} S_{mn}(-ika,\eta _0) S_{mn}(-ika,\eta ) cos(m(\phi -\phi _0))) \\&\quad \times \bigg {[} R^{(1)}_{mn}(-ika,i\xi _<) R^{(3)}_{mn}(-ika,i\xi _>) -\frac{R^{(1)'}_{mn} (-ika,0)}{R^{(3)'}_{mn} (-ika,0)}R^{(3)}_{mn}(-ika,i\xi )R^{(3)}_{mn}(-ika,i\xi _0) \bigg {]} \end{aligned} \end{aligned}$$

Here, $N_{mn}$ is the normalization constant and $\varepsilon _m$ is 1 for $m=0$ and 2 for all other values. $k=\frac{2\pi }{\lambda }$ is wavenumber. $\xi _<$ is $min(\xi ,\xi _0)$ and $\xi _>$ is $max(\xi ,\xi _0)$. $S_{mn} (-ika,\eta )$ is the oblate spheroidal angular wave function and $R_{mn}^{(j)} (-ika,i\xi )$ is the oblate spheroidal radial wave function of the $j^{th}$ kind.

**Figure 2:**
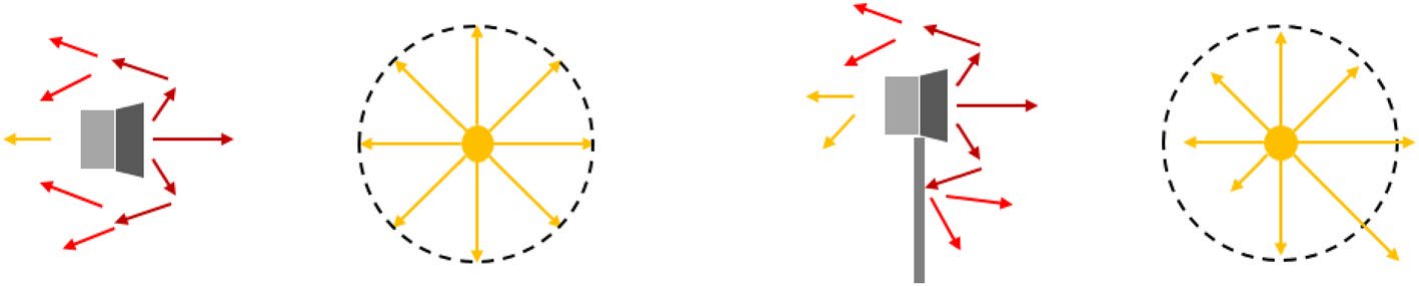
Configuration of the generated sound by a speaker in the free field (left) and a speaker with a barrier (right).

In the case of the control source, a circular control source is used in the prior study^13^. However, a circular control source which is an infinitely distributed monopole sources in a circle cannot consider the interaction between the control speakers and the barrier. The reason why control speakers are placed at the edge of the barrier is different from a circular source is illustrated in Fig. 2.

**Figure 3:**
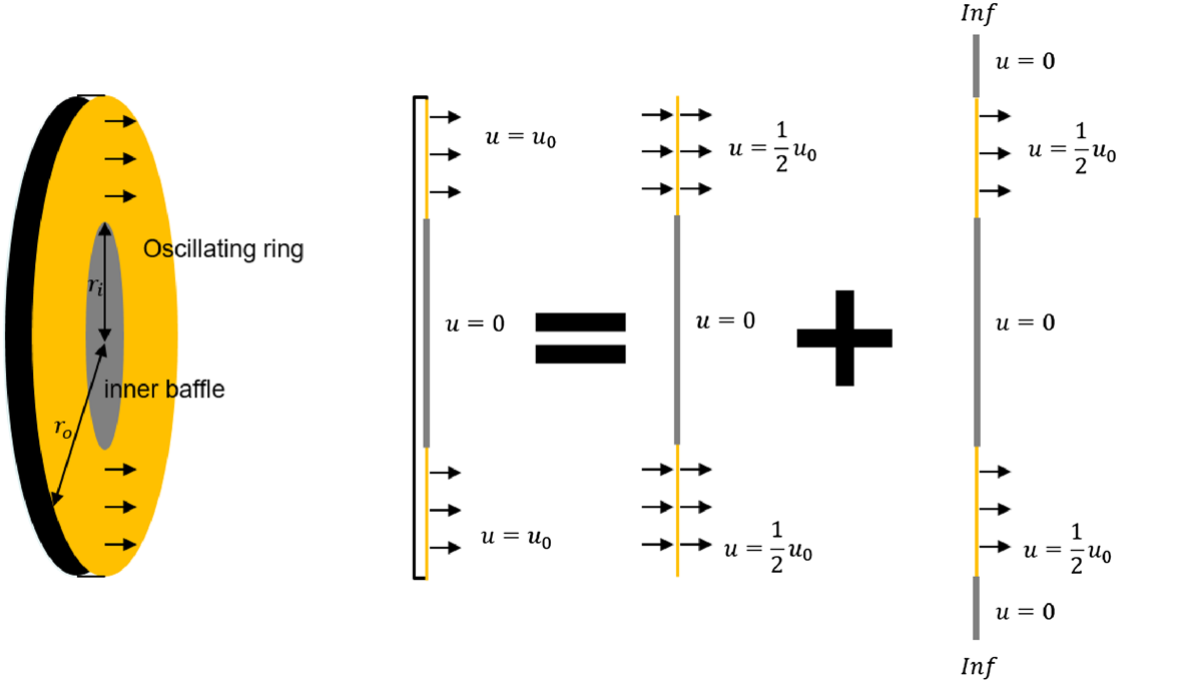
Configuration of an oscillating ring in a finite closed back baffle to consider the interaction between the speaker and the barrier.

As shown in Fig. 2, a speaker in the free field can be assumed as a monopole when a speaker is much smaller than the wavelength. However, a speaker attached to the edge of the barrier generates a different sound field compared with a monopole source because some of the sound propagating to the rear of the speaker is blocked by the barrier. To address this, an oscillating ring in a finite closed-back baffle is introduced to serve as a control source for considering the interaction between the speaker and the barrier. It is in a finite baffle with a closed-back, and the ring vibrates to generate sound, as shown in Fig. 3. The ring thickness ($r_o$-$r_i$) is determined by the diameter of the diaphragm of the control speaker.

Due to the structure of the vibrating piston with the internal baffle, an effect similar to the interaction between the speaker and the barrier appears, and therefore the case where the control speakers are placed on the edge of a circular barrier can be approximated. The equation of the oscillating ring in a finite closed-back baffle can be derived by changing the boundary condition of the oscillating disk in a closed-back baffle^17^. Compared to the circular control source, the model becomes more complex, but the axisymmetric property is maintained.

## Results

### Noise reduction performance in the simulation

**Figure 4:**
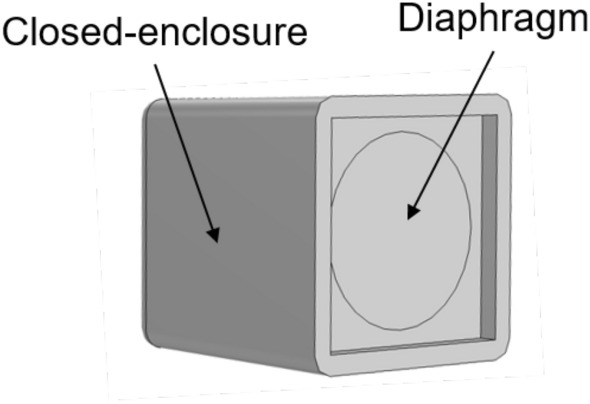
The speaker model used in the simulation is a closed-enclosure structure, and the sound is generated by oscillating the diaphragm.

The noise reduction performance of the proposed hybrid noise control system is checked through a FEM simulation by Comsol. In the simulation, the material of the barrier is selected as aluminum, and six simple speaker models are arranged on the edge of the barrier. The simple speaker model used in the simulation is shown in Fig. 4. It is a closed-enclosure structure, and the inside is filled with air. The sound is generated by setting the velocity of the diaphragm.

**Figure 5:**
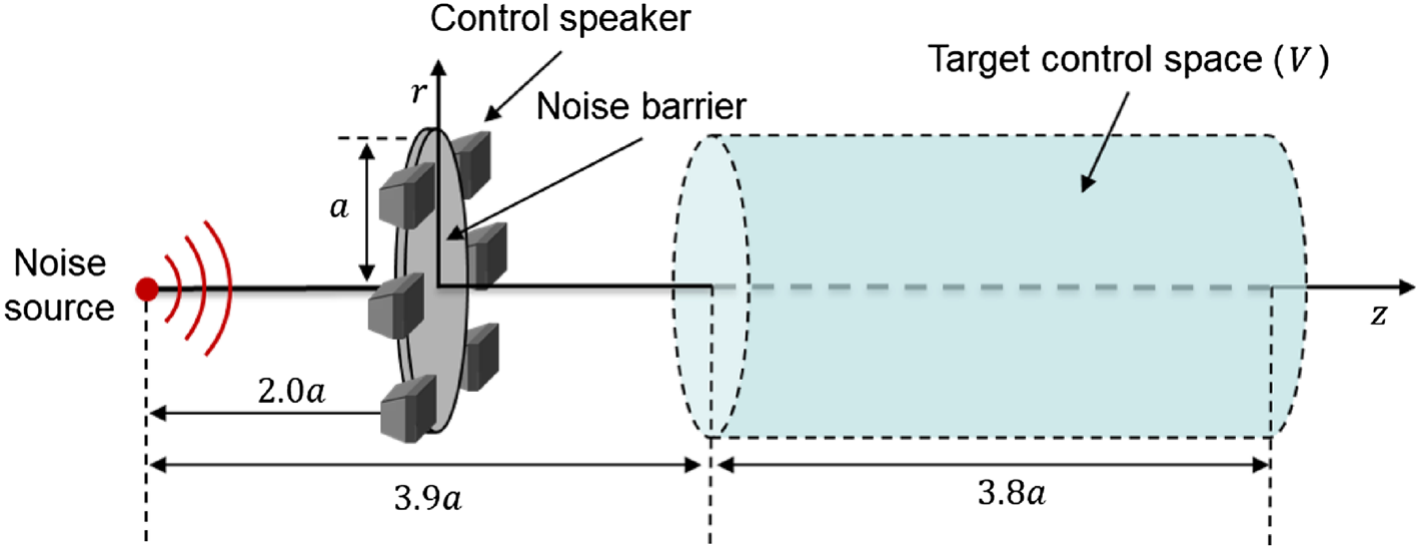
Configuration of the simulation model. A simple speaker model is used for control sources. a is the radius of the barrier. V is the target control space.

The speakers are placed so that the center of the speaker is on the edge of the barrier. The configuration of the simulation model is illustrated in Fig. 5.

**Figure 6:**
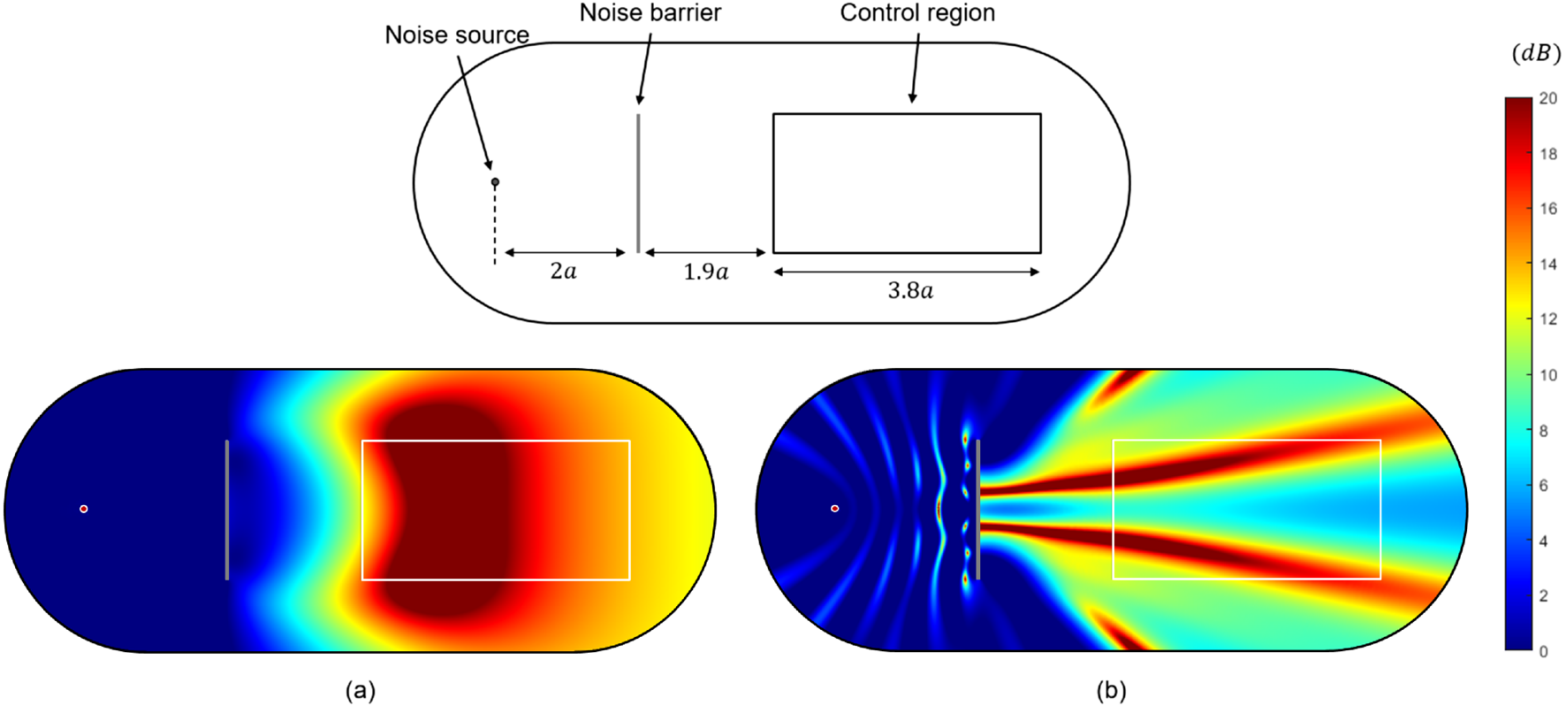
The cross-section of the simulation model on the $r-z$ plane (top). Insertion loss on the $r-z$ plane: (**a**) $ka=0.95$ and (**b**) $ka=9.5$.

The insertion loss on the $r-z$ plane is shown in Fig. 6 to confirm whether the noise is reduced in the control space. The insertion loss is defined as Eq. (9). $P_{n,0}$ is the pressure when the noise reduction methods are not applied, and $P_e$ is the residual pressure reduced by using the circular active barrier. $k=\frac{2\pi }{\lambda }$ is the wavenumber.

9 $$\begin{aligned} Insertion loss: 10 \log _{10} \bigg {[}\frac{P_{n,0}}{P_e}\bigg {]}^2 \end{aligned}$$

It is shown that the noise around the control space is reduced. Noise in a space opposite the control space may increase due to the control sound field for noise reduction in the target control space. If it is required to prevent the increase in noise over the space where the noise source is located, using a unidirectional control speaker can be one solution.

**Figure 7:**
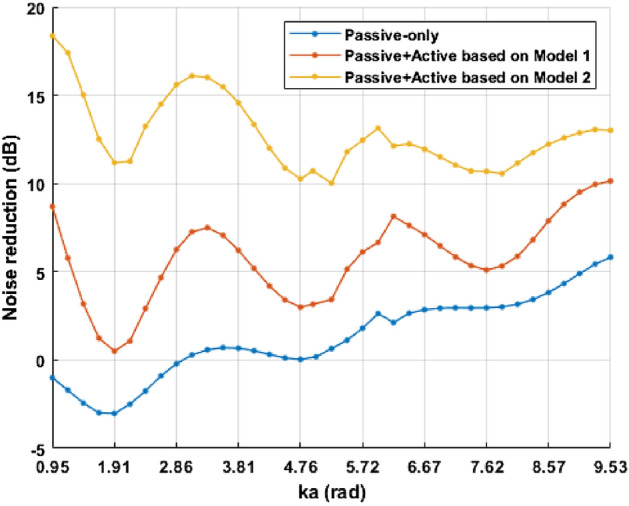
Noise reduction of circular barrier (blue), circular active noise barrier using the control filter obtained through the circular control source (red), and circular active noise barrier using the proposed theoretical model-based control filter (yellow).

The noise reduction defined in Eq. (5) is shown in Fig. 7. Theoretical model 1 is the case of using a circular source as a control source, and the proposed theoretical model 2 uses the oscillating ring in a finite closed-back baffle as a control source.

It is shown that the circular active noise barrier can reduce noise in the wide frequency band. However, it is shown that there is a performance gap between the two cases where different theoretical model-based control filters are used. The control filter based on the oscillating ring in a finite closed-back baffle model achieves better noise reduction than the control filter obtained by using a circular control source due to the interaction between the speaker and the barrier. As a result, the validity of the oscillating ring in a finite closed-back baffle as a control source in the theoretical model of the circular active noise barrier is confirmed.

### Experiments

#### Experimental set-up

**Figure 8:**
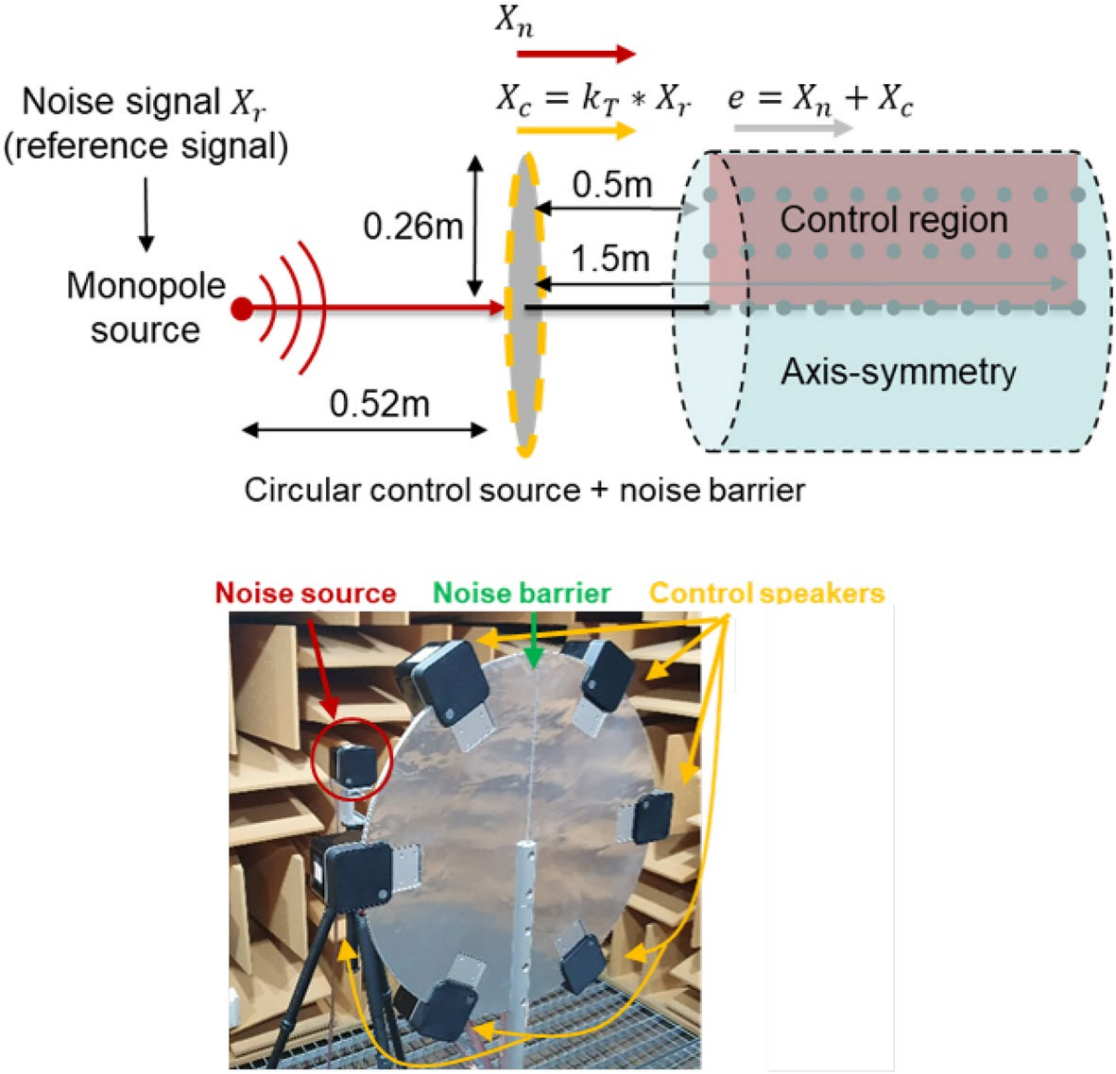
Configuration of the experimental system (top) and the constructed experimental system in an anechoic chamber (bottom).

The barrier was made of aluminum with a thickness of 6 *mm* and a density of 2.7$g/cm^3$. The radius of the barrier is 0.26 *m*. The speakers are placed so that the center of the speaker is on the edge of the barrier. The experiment was conducted in an anechoic chamber with a width and length of 3.6 *m*, a height of 2.4 *m*, and a minimum allowable frequency of 100 *Hz*. As noise and control sources, commercial speakers capable of generating a 200 to 20 *kHz* band were used. In the case of the frequency band of interest, the band from 200 *Hz*, the lowest frequency that the speaker can generate, to 2000 *Hz*, where the noise barrier can achieve noise reduction of 5 *dB* or more, is determined. Measurements were performed at intervals of 10 *cm* in one section of the target control space. The sampling frequency is 6000 *Hz* and Gaussian white noise was used as the noise signal. Open loop ANC is conducted without error microphones using the theoretically calculated control filter. In the case of the control filter, the time domain control filter obtained by inverse Fourier transform of the control filter in the frequency domain was used. The constructed experimental system is shown in Fig. 8. The globe experimental setup is the same as the previous study^13^.

#### Experiment results

**Figure 9:**
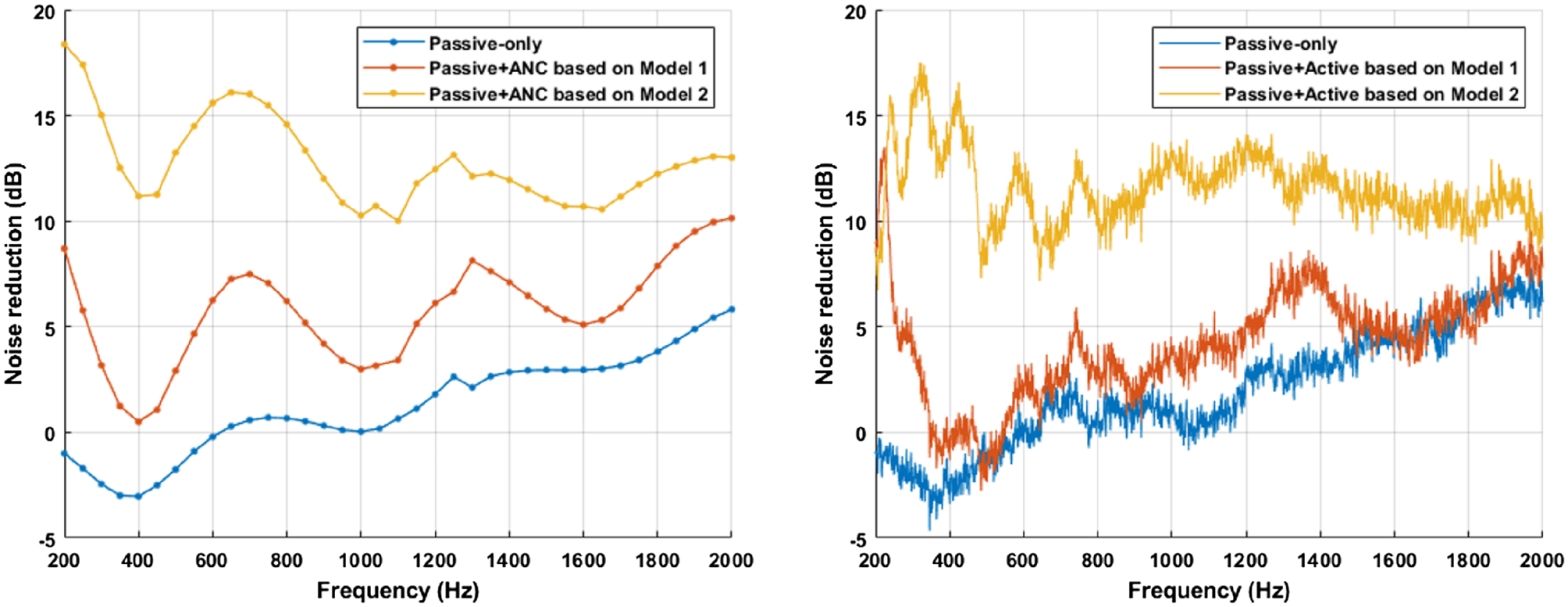
Noise reduction performance in the simulation (left) and in the experiment (right): noise barrier (blue), hybrid noise control using the control filter based on model 1 (red), and hybrid noise control using the control filter based on model 2 (yellow).

The measured noise reduction as defined in Eq. (5) is shown in Fig. 9. Theoretical model 1 is the case of using a circular source as a control source, and the proposed theoretical model 2 uses the oscillating ring in a finite closed-back baffle.

Similar to the simulation results, the circular noise barrier attenuates noise by about 5dB or more above 2000Hz, but it is shown that the noise reduction is insignificant or the noise is amplified below 1000Hz. In the hybrid noise control system, performance is improved by applying ANC. However, in the case of model 1, a difference in the control sound field occurs due to the interaction between the speakers and the barrier, resulting in poor performance. Otherwise, in the case of model 2, the hybrid noise control system achieves a noise reduction of about 10.6dB in the frequency band of interest by using the theoretically calculated control filter. The noise reduction performance as defined in Eq. (6) is shown in Table 1. As a result, it is validated that the oscillating ring in a finite closed-back baffle is more appropriate for the circular active noise barrier than a circular source.

**Table 1:** Noise reduction performance in the experiment.

	Noise reduction performance
Circular barrier	1.3 dB
Hybrid noise control (model 1)	3.6 dB
Hybrid noise control (model 2)	10.6 dB

## Discussion

**Figure 10:**
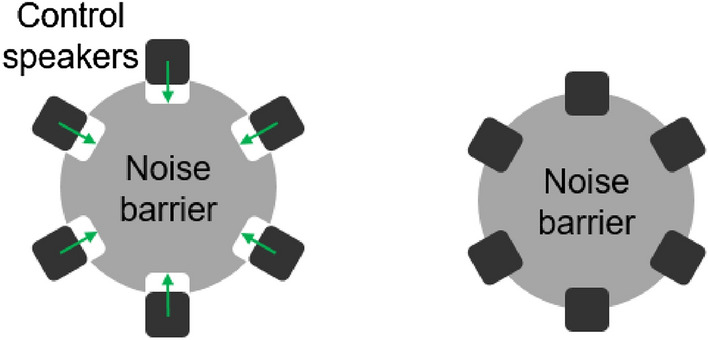
Configuration of the position of the control speakers.

According to the results in Fig. 9, it is shown that when model 1 is applied, the worse performance is achieved compared to the previous results^13^. The reason is that the placement position of the control speaker is moved inside for better performance. In an actual system, noise is scattered due to the arranged control speakers. However, the volume of the control speaker is not considered when theoretically calculating the sound field of noise, so the performance is degraded due to the difference in the sound field of noise. In order to reduce the effect of the volume of the control speaker, in this study, the speakers are placed so that the center of the speaker is on the edge of the barrier as shown in Fig. 10. Since the control speakers are moved inside the barrier, the interaction between the speakers and barrier becomes more significant. Therefore, when model 1 including a circular control source is applied, worse performance is achieved.

**Figure 11:**
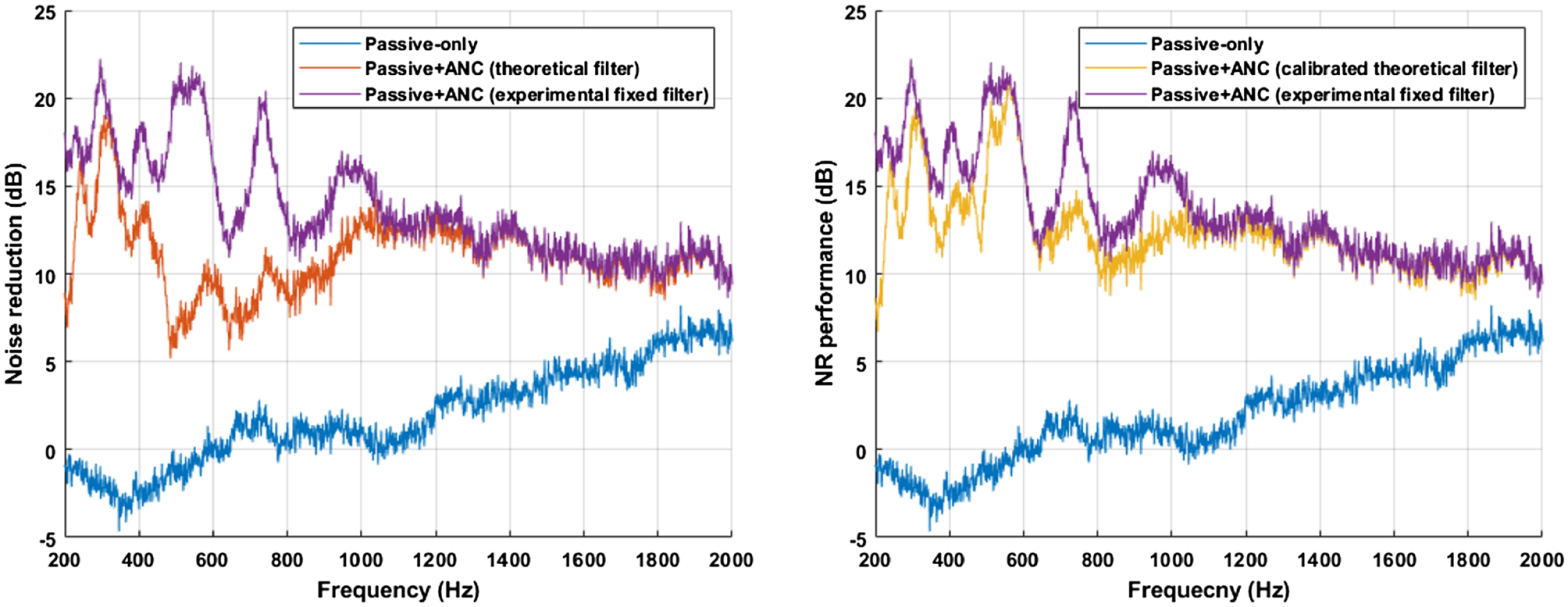
Noise reduction in the experiment: through the barrier only (blue), circular active noise barrier based on model 2 (red), circular active noise barrier using the calibrated theoretical control filter based on the preliminary experiment (yellow), and circular active noise barrier using the Wiener filter solution (purple).

For maximum performance of the constructed experimental system, the FIR Wiener filter solution^18^ obtained by the measured data over the target control space is used. The measured noise reduction as defined in Eq. (5) is shown in Fig. 11. The noise reduction performance as defined in Eq. (6) is 12.8 *dB*, constituting a difference in the performance of about 2.2 *dB* compared to the case of applying the theoretical model-based control filter.

**Figure 12:**
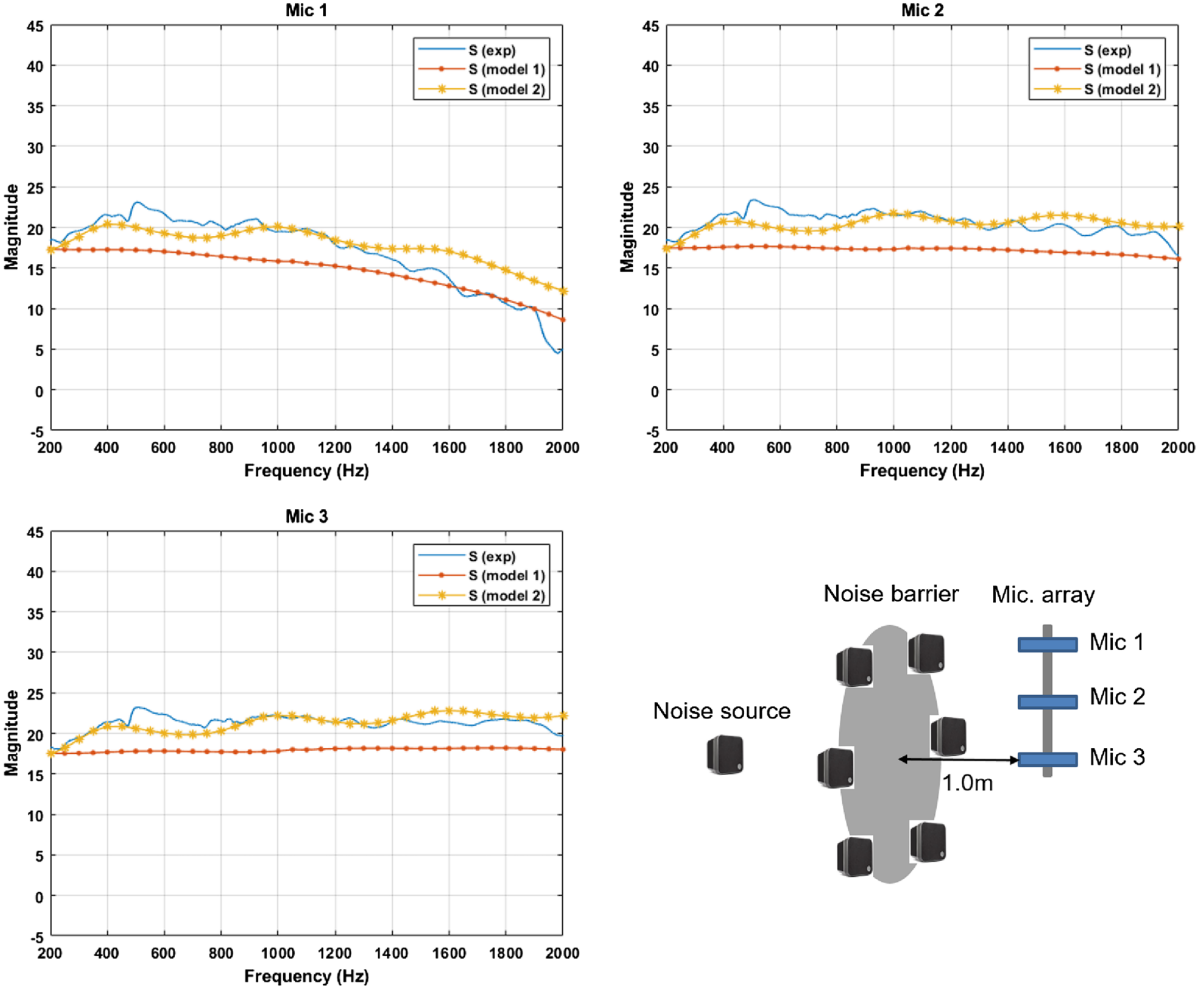
The secondary path at a distance of 1 m from the barrier: in the experiment (blue), model 1 (red), model 2 (yellow).

The noise reduction by using the theoretical control filter is lower than the case of the Wiener filter solution in a range of 450-900 *Hz*. A noticeable difference in the secondary path below 1000 *Hz* occurs between the theoretical model and the experimental system as shown in Fig. 12.

For performance improvement, the theoretical filter should be calibrated to consider the system dynamics, which are not included in the theoretical model. Noise reduction is shown in Fig. 11 when calibration based on the preliminary experiment in the anechoic chamber is applied. It is shown that the performance approaches that of the case of the Wiener filter solution.

## Conclusions

A circular active noise barrier that considers the interaction between a speaker array and a barrier is proposed to improve the noise reduction performance. In order to reduce the scattering of the noise due to the control speakers, the position of the control speaker is moved inside the barrier. This causes the influence of the interaction between the control speakers and a barrier to becoming more significant. Therefore, the oscillating ring in a finite closed-back baffle is introduced for the control source and the improvement of the noise reduction performance is validated through a simulation and experiment. It is confirmed that noise reduction similar to the experimental maximum performance could be achieved by using the theoretical control filter when the control filter is tuned based on a preliminary experiment. Further research such as studying methods of obtaining the required information for ANC or the means of updating the control filter should be carried out.

## Data Availability

The datasets generated and analyzed during the current study are available from the corresponding author on reasonable request.
